# Supplementary material for: Disentangling entanglements in biopolymer solutions
Source: Nat Commun. 2018 Feb 5;9:494. doi: 10.1038/s41467-018-02837-5 (PMC5799238; doi:10.1038/s41467-018-02837-5)
Supplement: Supplementary file 1 — Supplementary Information [file 41467_2018_2837_MOESM1_ESM.pdf]

SUPPLEMENTARY FIGURE 1

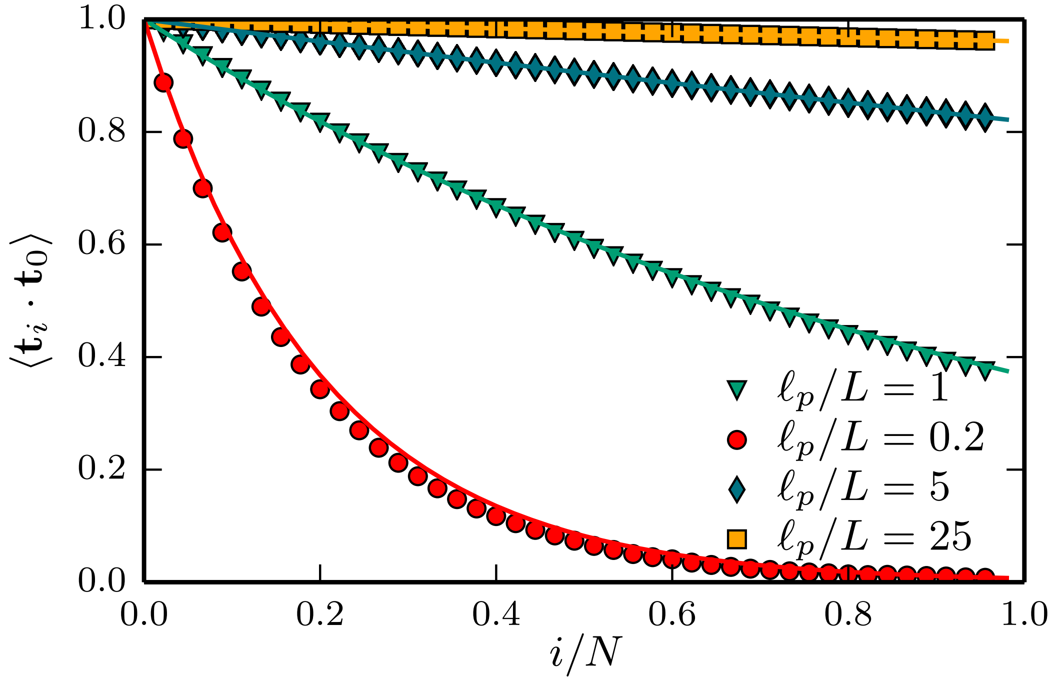

Supplementary Figure 1. **Tangent-tangent correlation function** of a polymer in an entangled solution with mesh size $\xi/L = 0.14$ for different values of the relative persistence length ℓ_p/L indicated in the graph. The theoretical prediction, Eq. (1), (solid lines) agrees very well with the numerical results (closed symbols). Statistical errors are about the symbol size.

SUPPLEMENTARY FIGURE 2

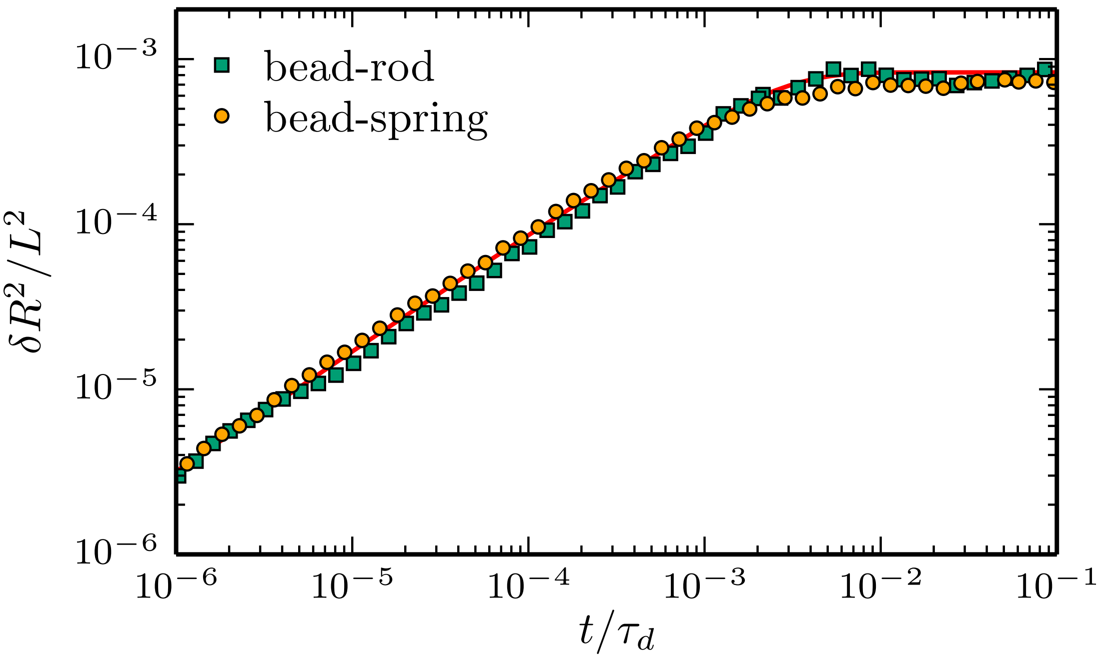

Supplementary Figure 2. **Time evolution of the end-to-end distance fluctuations** $\delta R^2(t)$ of the weakly-bending limit for the worm-like chain model (solid red line, Eq. (2)) is shown together with the results from Brownian dynamics simulations. We show the results of the bead-spring algorithm used in this work (solid yellow circles) and compare it with the theoretical prediction (Eq. (2)) and simulations employing a bead-rod algorithm (green solid squares) [1, 2]. In the bead-spring case, the numerical data were obtained for polymers with $\ell_p/L = 5$ in a rather dilute solution with $\xi/L = 0.34$. The bead-rod algorithm employs free polymers of identical characteristics. The deviations between simulations and between simulations and analytical results are all comparable to the statistical error which are about symbol size.

SUPPLEMENTARY FIGURE 3

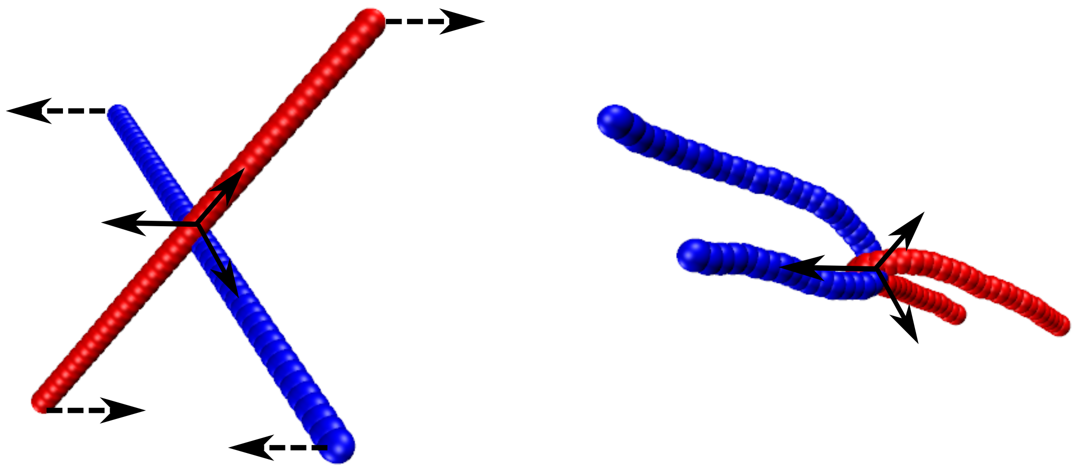

Supplementary Figure 3. **Setting used to test for chain crossings**. The initial configuration is shown on the left, a typical snapshot of a state during the simulation on the right. The coordinate system is given by the arrows, the pulling forces are indicated by the dashed arrows.

SUPPLEMENTARY FIGURE 4

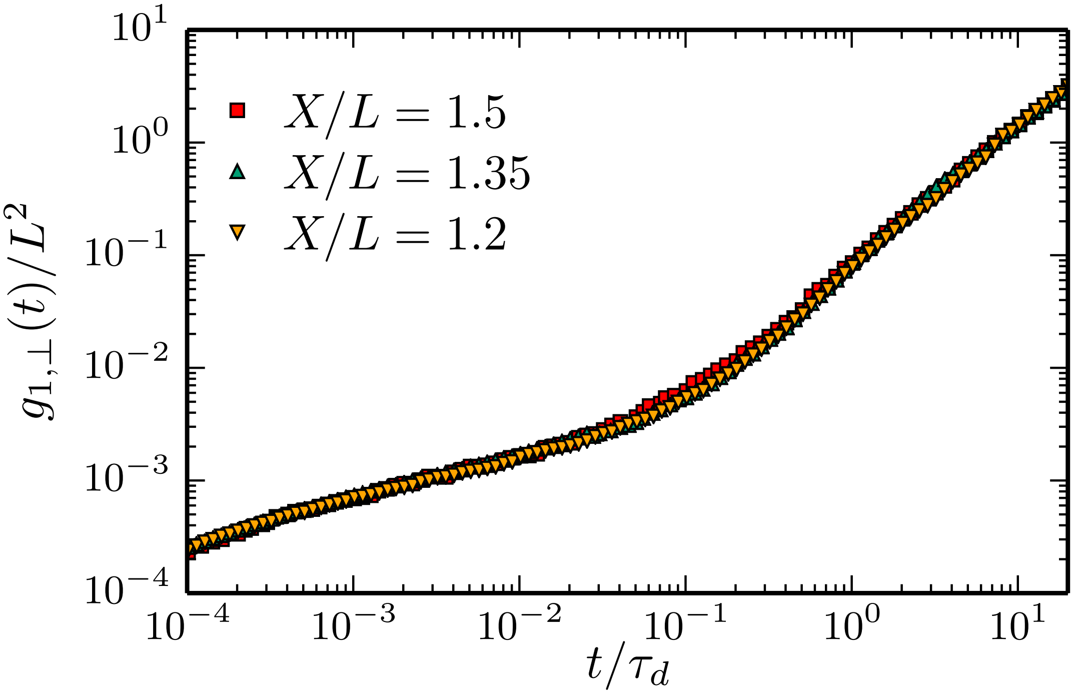

Supplementary Figure 4. **MSD of the center monomer perpendicular to the orientation of the end-to-end vector** $g_{1,\perp}(t)$ in a polymer solution with $\xi/L = 0.086$ and $\ell_p/L = 1$ for different sizes, X/L, of the simulation volume as indicated in the graph.

SUPPLEMENTARY FIGURE 5

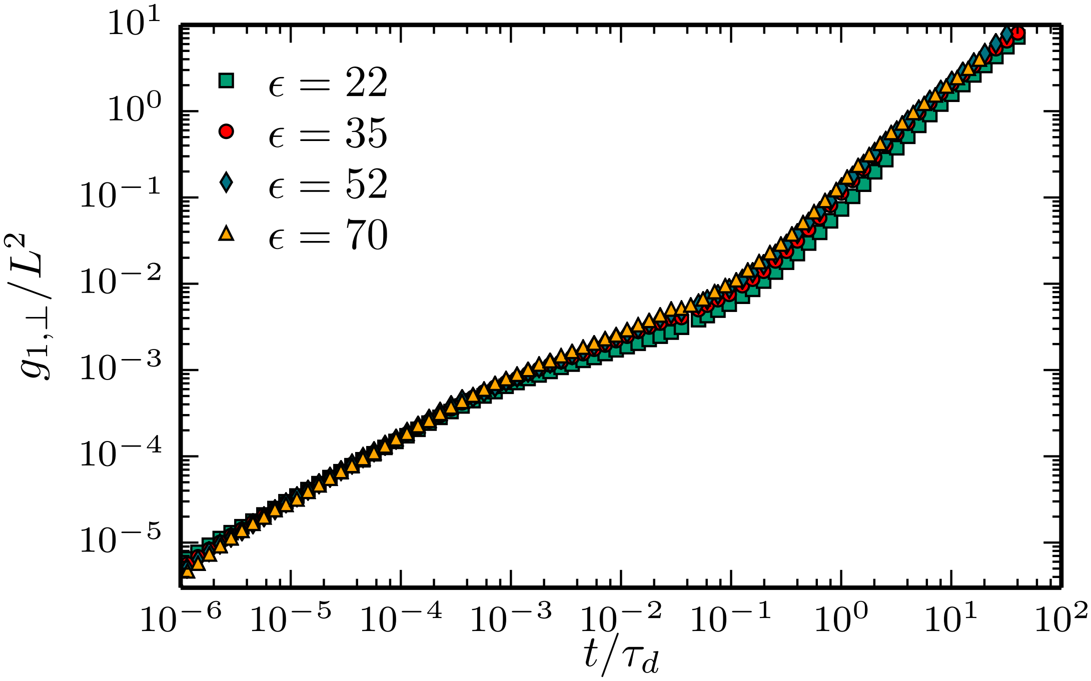

Supplementary Figure 5. **MSD of the center monomer perpendicular to the orientation of the end-to-end vector** $g_{1,\perp}(t)$ in an entangled polymer solution with $\xi/L = 0.17$ and $\ell_p/L = 25$ for different aspect ratios $\epsilon = L/\sigma$ as indicated in the graph.

SUPPLEMENTARY FIGURE 6

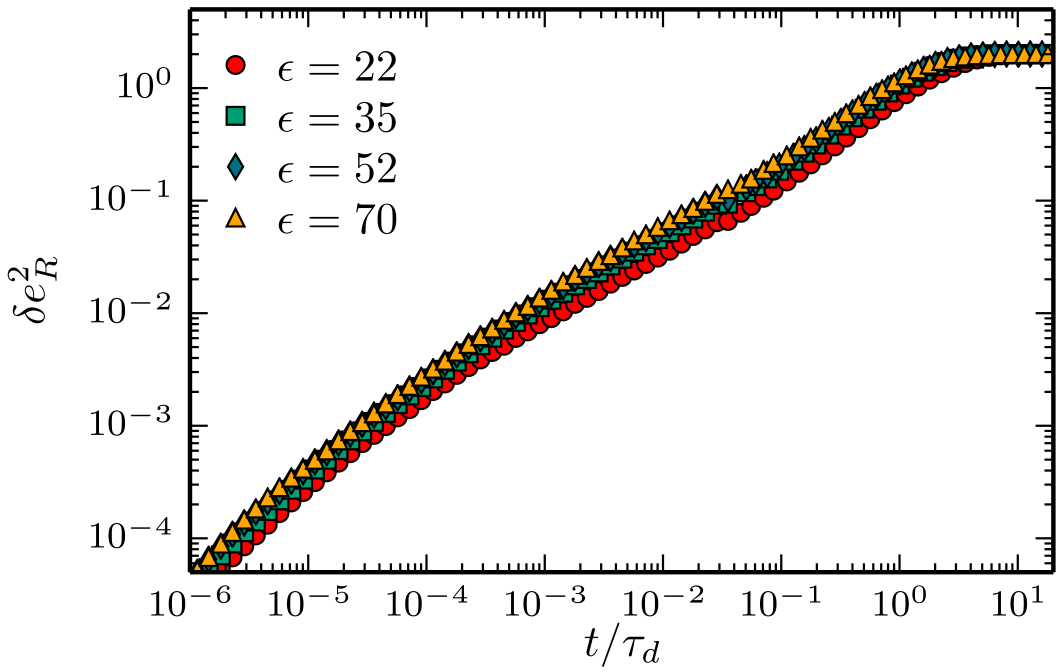

Supplementary Figure 6. **MSD of the orientation of the end-to-end vector** $\delta e_R^2(t)$ of a tracer polymer in an entangled polymer solution with $\xi/L = 0.12$ and $\ell_p/L = 1$ for different aspect ratios $\epsilon = L/\sigma$ as indicated in the graph.

SUPPLEMENTARY FIGURE 7

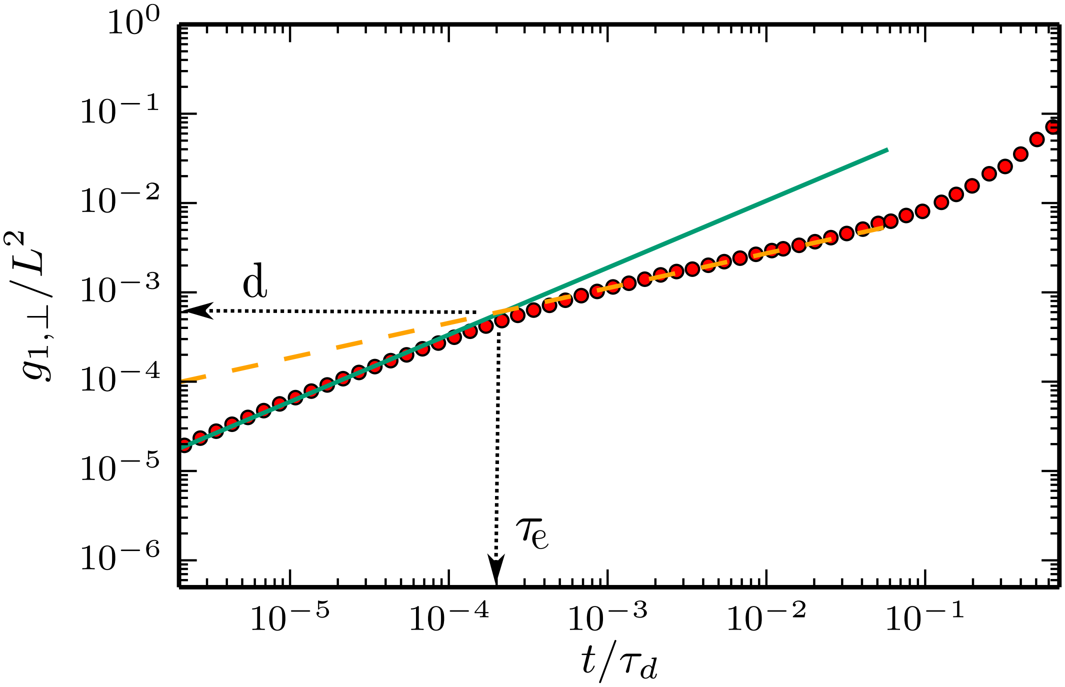

Supplementary Figure 7. **Definition of the tube diameter.** The time evolution of $g_{1,\perp}(t)$ exhibits a $t^{3/4}$ regime at early times (solid green line), followed by an intermediate regime, where a polymer's bending fluctuations are hindered by the surrounding polymers. We fit this intermediate regime with a effective power law (dashed yellow line). The intersection of the dashed yellow line and the $t^{3/4}$ regime (solid green line) defines the tube diameter as well as the corresponding entanglement time τ_e. The data in this plot are for an entangled polymer solution with $\xi/L = 0.1$ and $\ell_p/L = 1$.

SUPPLEMENTARY FIGURE 8

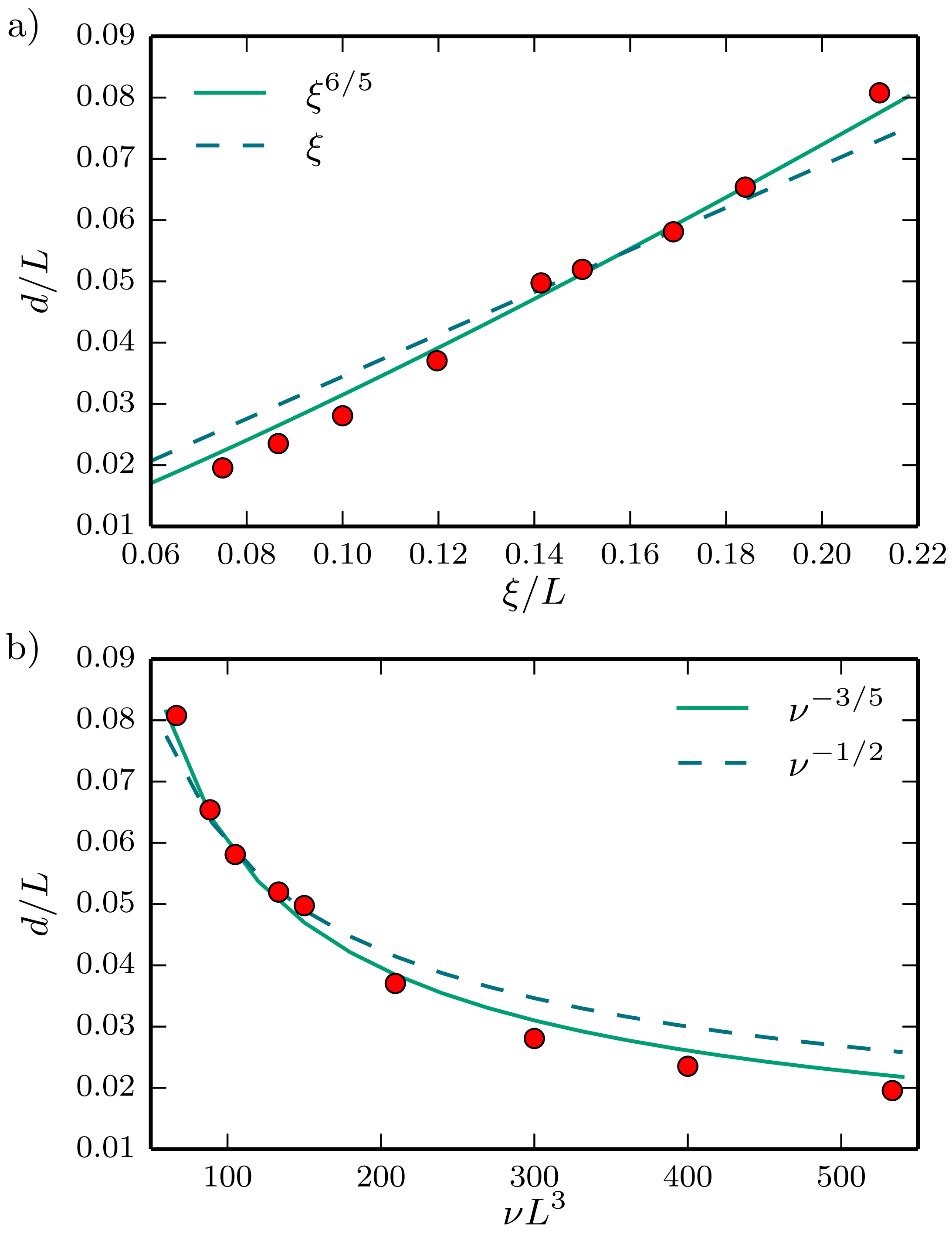

Supplementary Figure 8. **Tube diameter** d/L as a function of a) mesh size ξ/L, and, equivalently, b) the number density $\nu L^3 = 3L^2/\xi^2$ as obtained from the analysis illustrated in Supplementary Fig. 7 for polymers with $\ell_p/L = 1$. The data points (filled red circles) are well fitted by scaling laws $\xi^{6/5}$ and $\nu^{-3/5}$ (solid green lines), respectively. For comparison, the blue dashed lines show a tube diameter scaling linearly with the mesh size, $d \sim \xi$, which does not fit our data as well as the Odijk-Semenov scaling laws.

SUPPLEMENTARY FIGURE 9

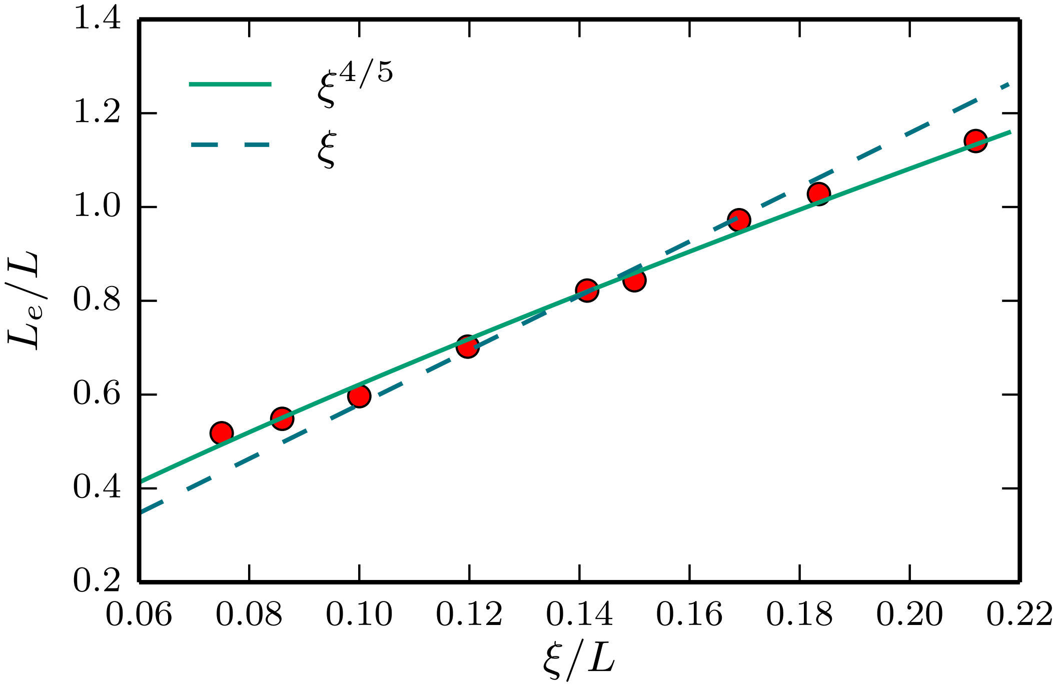

Supplementary Figure 9. **Entanglement length** L_e/L as a function of the mesh size ξ/L for polymers with $\ell_p/L=1$. The simulation data (filled red circles) are consistent with $L_e \sim \xi^{4/5}$ (solid green line) and deviate significantly from $L_e \sim \xi$ (dashed blue line).

SUPPLEMENTARY FIGURE 10

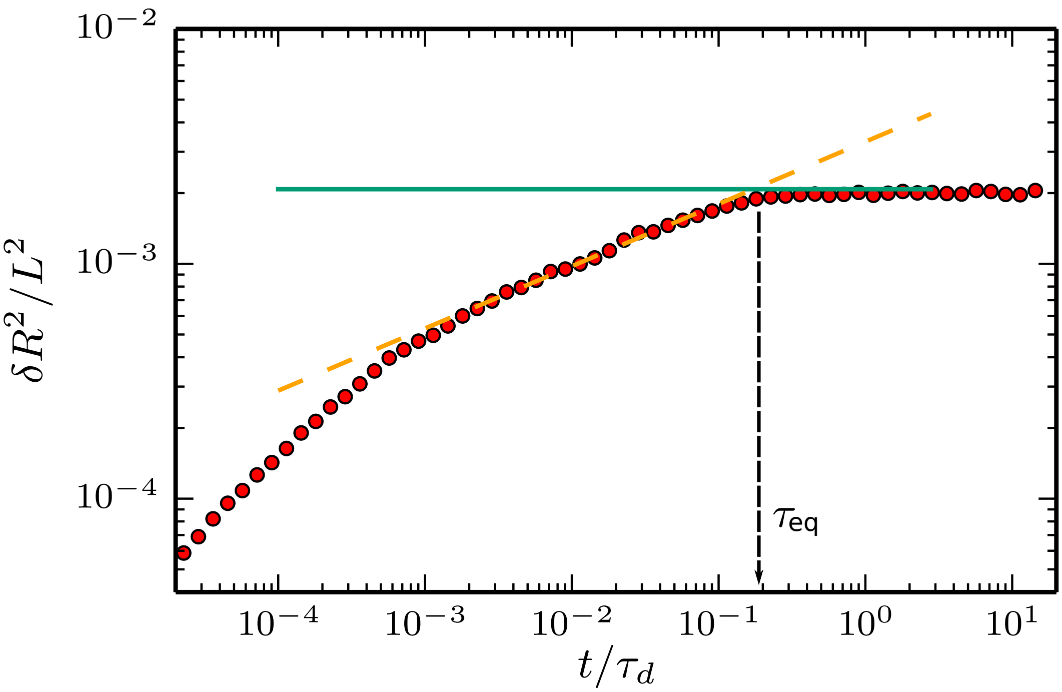

Supplementary Figure 10. **Determination of the equilibration time** τ_{eq}. The crossover from the intermediate regime to saturation of the contour fluctuations is used to determine τ_{eq}. The solid green line shows the saturation value of the internal relaxation. The dashed yellow line represents an effective power law fitted to the intermediate regime. The intersection of the dashed yellow and solid green line is used to obtain the value of τ_{eq} (see dashed black line). The simulation data in this figure are for an entangled solution with $\xi/L = 0.086$ and $\ell_p/L = 3$.

SUPPLEMENTARY FIGURE 11

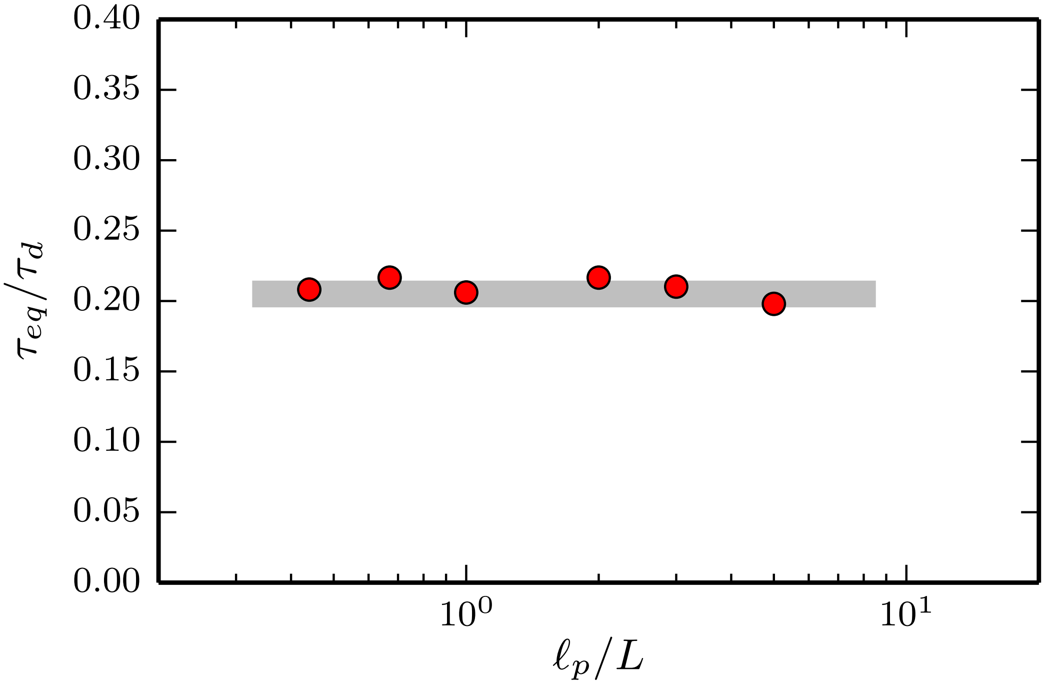

Supplementary Figure 11. **Equilibration time of internal modes** τ_{eq} as a function of the persistence length ℓ_p for an entangled solution with mesh size $\xi/L = 0.086$. As indicated by the horizontal grey bar, no effect of the polymer stiffness on the equilibration time τ_{eq} is noticeable; data points are given by filled red circles.

SUPPLEMENTARY FIGURE 12

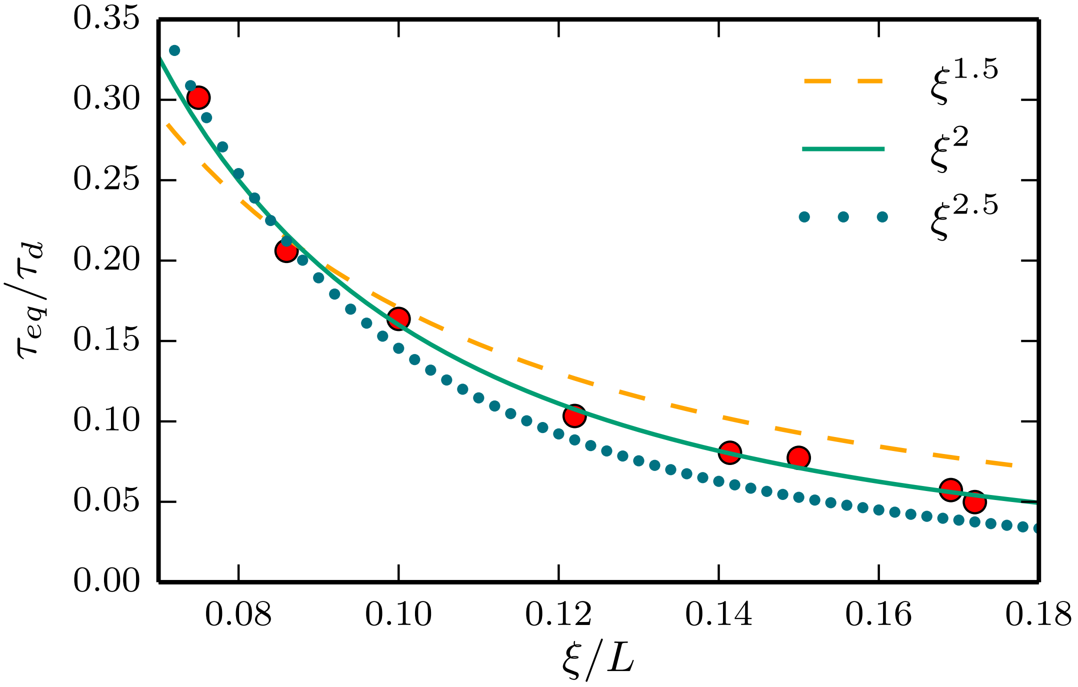

Supplementary Figure 12. **Mesh size dependence of the equilibration time** τ_{eq} for a representative system with $\ell_p/L = 1$. Among the various power laws, $\tau_{eq} \sim \tau_d/\xi^2$ (solid green line) fits the numerical data (filled red circles) best.

SUPPLEMENTARY FIGURE 13

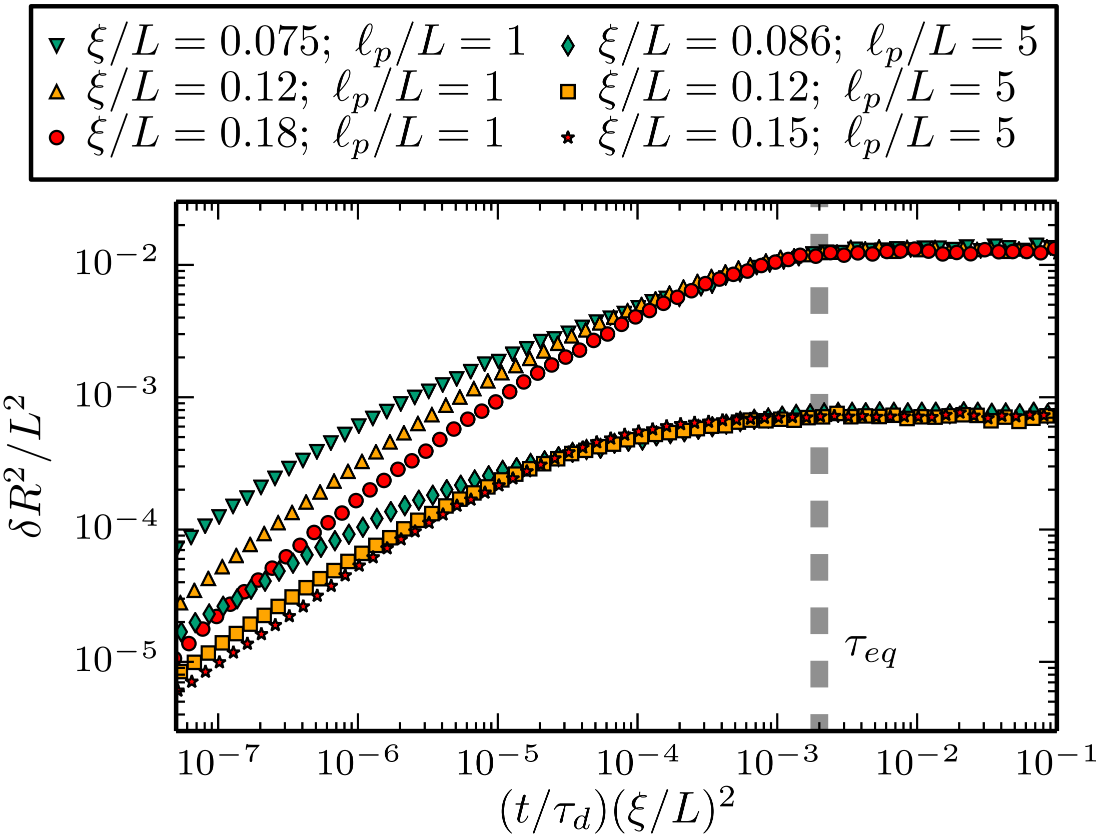

Supplementary Figure 13. **Data collapse of the end-to-end distance fluctuations.** Upon rescaling time by $\tau_d\,(L/\xi)^2$ we find excellent data collapse in the terminal relaxation regime for two different persistence length, $\ell_p/L = 1$ and $\ell_p/L = 5$, and a range of mesh sizes ξ/L as indicated in the graph.

SUPPLEMENTARY FIGURE 14

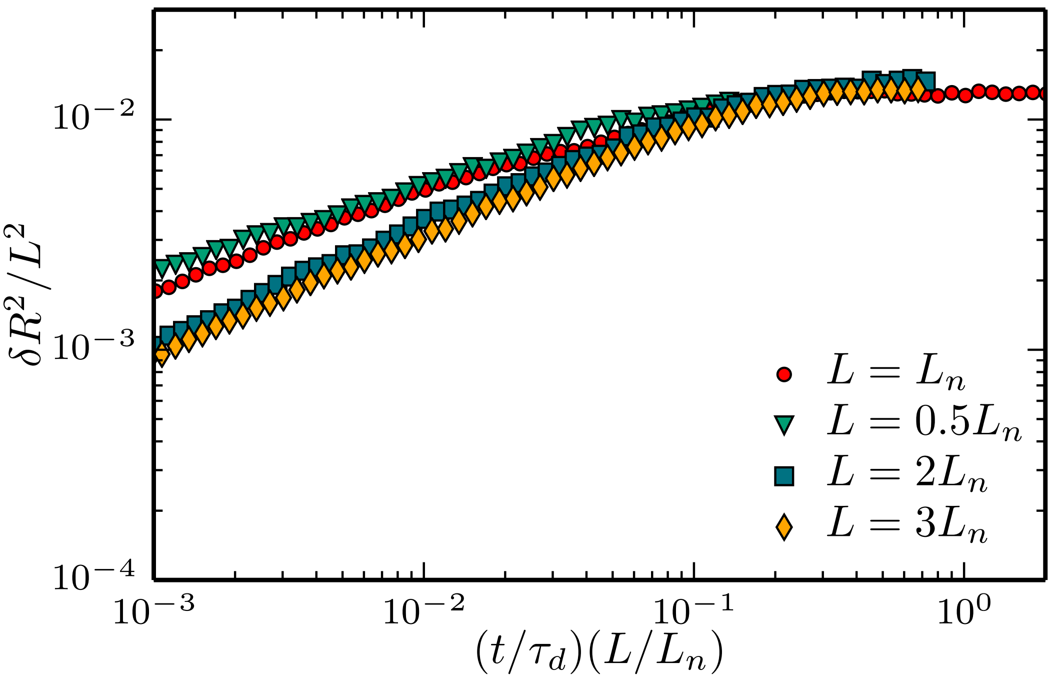

Supplementary Figure 14. **Scaling of the equilibration time of a tracer filament:** $\delta R^2(t)$ for a tracer polymer of length L embedded in an entangled solution of polymers with contour length L_n, and mesh size $\xi/L = 0.086$; all polymers have a persistence length $\ell_p = L$. Good data collapse is achieved by rescaling time with $\tau_\times \sim \tau_d L_n/L \sim L^2 L_n$ instead of the time scale suggested by a classical constraint release mechanism, $\tau_\times^{cr} \sim L_n^3$; relative values of L/L_n are indicated in the graph.

SUPPLEMENTARY FIGURE 15

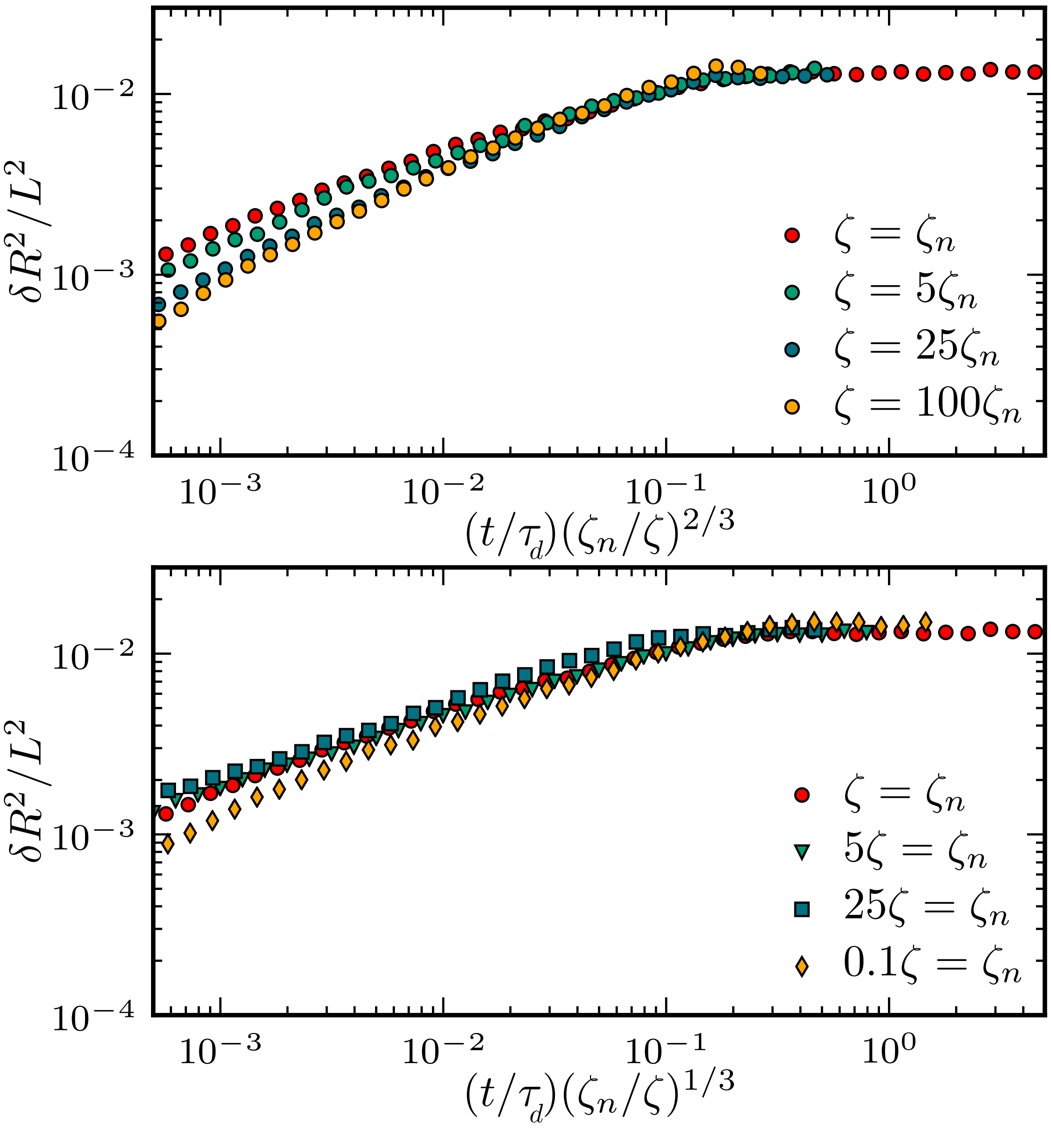

Supplementary Figure 15. **Scaling of the mean-square end-to-end distance with friction coefficients.** A single polymer with a friction coefficient ζ is considered in an entangled solution of polymers whose friction coefficient ζ_n differs from ζ. Simulations were performed for polymers with $l_p/L = 1$ in solutions with a mesh size $\xi/L = 0.086$, and a range of values for ζ_n and ζ as indicated in the graph. In the upper panel, the friction coefficient of the surrounding polymers is fixed, $\zeta_n = 1$, and ζ is varied, and vice versa in the lower panel. Upon changing the friction of the tracer filament, rescaling the time with $(\zeta_n/\zeta)^{2/3}$ results in a good collapse of the internal relaxation time. Consistently using $(\zeta_n/\zeta)^{1/3}$ achieves a data collapse, if the friction coefficient of the surrounding filaments is altered.

SUPPLEMENTARY FIGURE 16

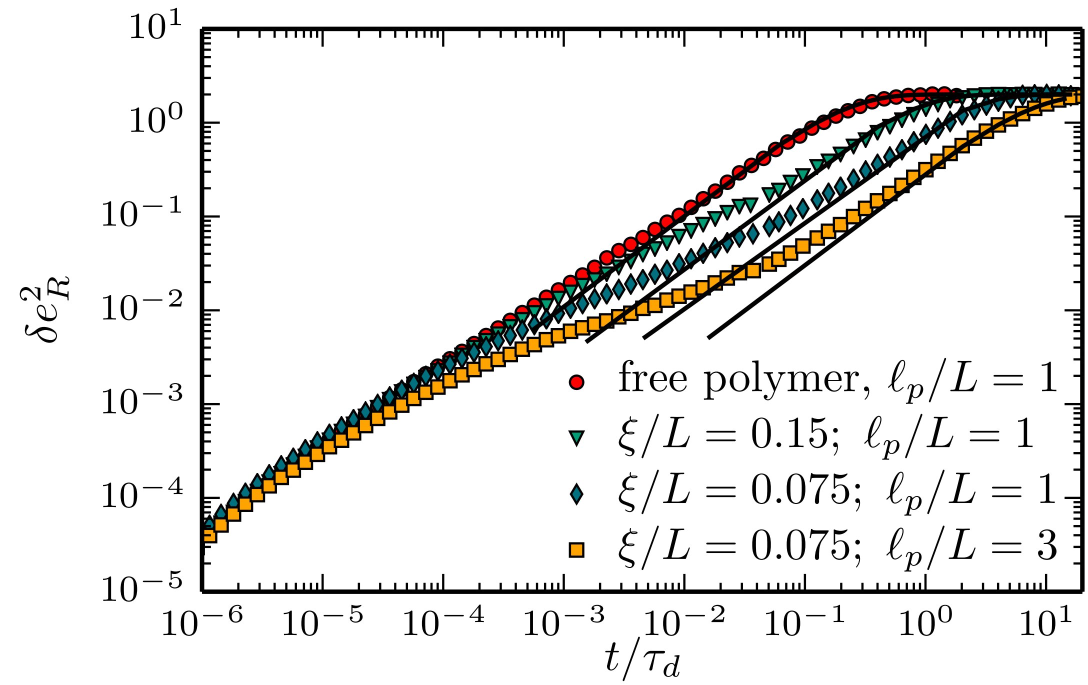

Supplementary Figure 16. **Dynamics of rotational relaxation.** MSD of the orientation of the end-to-end vector $\delta e_R^2(t)$ for different polymer stiffnesses ℓ_p/L and mesh sizes ξ/L as indicated in the graph. The terminal dynamics is described well by the same functional form as valid for the free relaxation of a rigid rod in solution: $2 - 2\exp(-t/\tau_r)$; see solid black lines.

SUPPLEMENTARY FIGURE 17

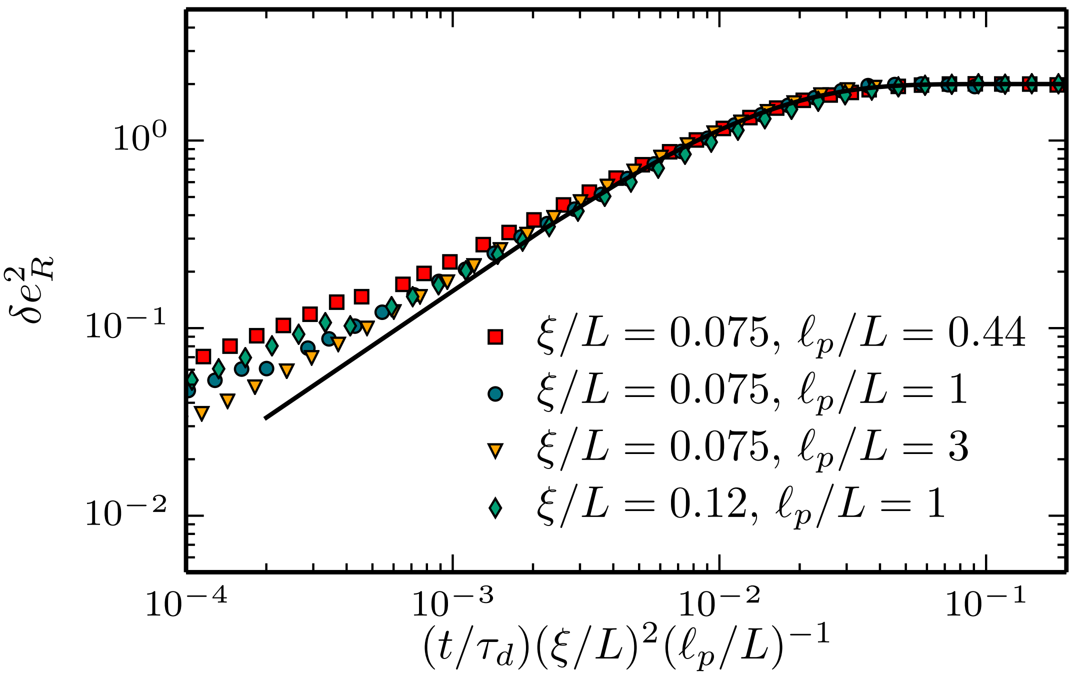

Supplementary Figure 17. **Dynamics of the rotational relaxation.** Scaling of the terminal relaxation time in the semiflexible regime. With the new scaling law proposed in the main text, a good data collapse onto an universal $2 - 2\exp(-t/\tau_r)$ behaviour (black line), almost through the full $2 - 2\exp(-t/\tau_r)$ regime, is achieved.

SUPPLEMENTARY NOTE 1: TESTS OF BROWNIAN DYNAMICS SIMULATIONS

We performed extensive tests to ensure the reliability of our Brownian dynamics simulations. First, we tested some known static quantities, in particular the tangent-tangent correlation function of an individual polymer chain. Then we tested the dynamics of the system by analysing the results of our simulation in dilute solution. Finally, we analysed the effect of system size and filament thickness on the dynamics to find parameter ranges where these have only negligible effects on the dynamics of the entangled polymer solution.

Tangent-tangent correlation function. One of the most basic quantities characterising the statistical properties of the worm-like chain model is the tangent-tangent correlation function which is given by [3, 4]

$$\langle \mathbf{t}_i \cdot \mathbf{t}_j \rangle = \exp(-|j-i|/\ell_p)\,, \tag{1}$$

where ℓ_p denotes the polymer's persistence length. As shown in Supplementary Fig. 1, our simulations agree very well with the expected exponential decay, both for dilute and a dense entangled polymer solutions.

End-to-end distance fluctuations. A known possible issue of bead-spring algorithms like the one used in this work is the finite extensibility of the springs. It can be avoided by using a constrained algorithm simulating an inextensible chain [1, 2, 5–7]. Alternatively, as done in this work, a proper choice of the computational parameters is necessary such that the finite extensibility only affects the dynamics at small times but leaves the dynamics at long times unchanged. To carefully check for effects from chain extensibility we simulated the dynamics of the end-to-end distance fluctuations $\delta R^2(t)$ in dilute solutions and tested it against known analytical results for free polymers [8–12]:

$$\delta R^2(t) = \frac{4}{\ell_p^2} \sum_n \left(\frac{L}{\pi n}\right)^4 \left[1 - \exp\left(-2\frac{\ell_p k_B T}{\zeta L^4}(\pi n)^4 t\right)\right]\,. \tag{2}$$

Additionally, we tested our simulations against the results of a bead-rod algorithm of free polymers [1, 7], which fully respects the inextensibility of the polymer chain and was implemented by our group as well [2]. As shown in Supplementary Fig. 2, there is excellent agreement between our Brownian dynamics simulations and both the analytical predictions and the bead-rod algorithm. Thus we conclude that on the timescales of interest our algorithm gives correct results within the error margin.

Test of chain crossing. In this work, we are interested in the dynamics of entangled solutions where topological constraints hinder the dynamics of the polymers. Therefore, it is of utmost importance to ensure that these constraints are faithfully implemented, and the Brownian dynamic simulations do not show any spurious chain crossings. In order to test that polymer chains do not cross in our simulations we studied the scenario illustrated in Supplementary Fig. 3: Two initially stretched chains are positioned in the simulation box to form a cross, one along the x-axis and the other parallel to the y-axis such that the center of both chains is on the z-axis. Initially, the shortest distance between the chains is set to $1.5\,\sigma$. We considered a situation where the ends (outermost beads) of these chains are pulled with a (spring) forces of magnitude $10\,k_BT/a_0$ against the other parallel to the z-direction; see arrows in Supplementary Fig. 3. These forces are larger than the thermal forces acting on a single bead, which are of order k_BT/a_0. In three runs of the Brownian dynamics simulations, which each lasted for a time period of $3\tau_d$, we did not find any chain crossings. Thus we concluded that the implementations of both the excluded volume and the connectivity of the chain

are chosen such that chain crossings are prevented. As an additional precaution, we included an abort condition in all of our simulations. If the distance between any two beads decreases below $0.75\,\sigma$ or the bond length in a chain increases above $1.2\,L/N$ the simulation was aborted with an error.

Simulation volume and finite size effects. The size of the simulation volume was determined such as to avoid finite size artefacts and still achieve acceptable run times (less than 14 days on an Intel Xeon E5-2670v2 CPU) for each individual simulation run. Supplementary Fig. 4 compares simulation results for the transverse dynamics of the center monomer for different values of X/L (indicated in the graph) for an exemplary system with $\ell_p/L = 1$ and density $\xi/L = 0.086$. We obtained similarly good agreement over many decades in simulation time for other observables, and concluded that $X = 1.35\,L$ is a good choice for the size of our simulation box. In particular, since $X > L$, this makes it impossible for one end of a polymer to come into contact with its other end.

Filament thickness (aspect ratio). Finally, we wanted to test for the effect of the choice of filament thickness on the dynamics of the entangled polymer solution. Since the mutual interaction between beads is given in terms of the WCA potential, we used the range σ of that potential as a measure for the filament diameter. For biopolymers like F-actin the ratio of filament length to filament thickness (aspect ratio) is approximately $L/\sigma \approx 10^3$. This value is significantly smaller compared to $L/\sigma \approx 10^2$ which we used in our simulations; note that the choice of the aspect ratio is dictated by the degree of discretisation of the polymer chain (number of beads N). Therefore, we tested how quantities like $g_{1,\perp}(t)$ and $\delta e_R^2(t)$ would depend on different choices of the filament's aspect ratio $\epsilon := L/\sigma$; see Supplementary Fig. 5 and Supplementary Fig. 6.

We performed simulations in two very different parameter ranges of the polymer stiffness for a range of aspect ratios ϵ. Supplementary Fig. 5 shows the dynamics of transverse contour fluctuations, $g_{1,\perp}(t)$, of a rather stiff tracer polymer with $\ell_p/L = 25$ in a solution with mesh size of $\xi/L = 0.17$, and Supplementary Fig. 6 shows the relaxation of a tracer filament's orientation, $\delta e_R^2(t)$, with $\ell_p/L = 1$ in a solution with mesh size $\xi/L = 0.12$. These simulations show that the dynamics depend only weakly on the choice of the aspect ratio (chain discretisation) once the aspect ratio becomes larger than 50. Therefore, for the results presented in this study, we used chains with $N = 45$ (corresponding to $L/\sigma \approx 52$) and for higher densities $N = 60$ (corresponding to $L/\sigma \approx 70$).

As there is hardly any dependence on the aspect ratio for systems with $\epsilon \gtrsim 35$, we concluded that we have reached the slender filament limit in our simulation, such that only the topology is relevant for the dynamics. Due to this observation, the mesh size ξ suffices to uniquely describe the system, whereas the aspect ratio or the occupied volume fraction is irrelevant for the dynamics of an entangled polymer solution, the aim of the current study. Especially, this strongly suggests that the results of our simulations remain valid for filaments of a much higher aspect ratio.

SUPPLEMENTARY NOTE 2: ODIJK-SEMENOV SCALING

For semiflexible polymers, Odijk and Semenov [13, 14], developed scaling theories to explain the effect of topological constraints on the bending modes of semiflexible polymers in entangled solutions. They proposed that these topological constraints are effectively described by a tube of diameter d which limits the amplitude of a polymer's bending fluctuations. Specifically, they predicted that the tube diameter should scale as $d \sim \xi^{6/5} \ell_p^{-1/5}$ [13, 14], and that the ensuing 'entanglement length' (collision length with the tube) should scale as $L_e^3 \sim d^2 \ell_p$ [13].

Since the tube is a phenomenological concept to characterize the *transient* effect of topological constraints, there is some degree of ambiguity in the definition of the tube diameter. If one wants to define it as the width of a polymer's bending fluctuations one has to define a proper time interval during which those fluctuations are sampled [15]. This time interval has to be of the order of the entanglement time $\tau_e \sim L_e^4/\ell_p$ [12], but is otherwise not well-defined. Therefore, we chose to define the tube diameter d as that value of the transverse fluctuations $g_{1,\perp}(t)$ where it starts to deviate from the $t^{3/4}$-behaviour of free polymers. As illustrated in Supplementary Fig. 7, we fitted the dynamics of $g_{1,\perp}(t)$ in the intermediate regime by an effective power law (dashed lines) and determined the intersection with the $t^{3/4}$ power law (solid line). This determines both the tube diameter d and the entanglement time τ_e, and with $\tau_e \sim L_e^4/\ell_p$ also the entanglement length L_e.

The resulting values of the tube diameter d for various values of the mesh size ξ are shown in Supplementary Fig. 8. We find good agreement with the scaling law predicted by Semenov and Odijk [13, 14], in accordance with results from earlier simulations [16] as well as experiments [15]. Given the expression for the tube diameter, the entanglement length may also be written as $L_e \sim \xi^{4/5} \ell_p^{1/5}$. We extracted the value for the entanglement length L_e from data as shown in Supplementary Fig. 7 using $\tau_e \sim L_e^4/\ell_p$. As shown in Supplementary Fig. 9, our data are again consistent with Odijk-Semenov scaling.

SUPPLEMENTARY NOTE 3: SCALING OF THE EQUILIBRATION TIME OF INTERNAL BENDING MODES

Scaling in a monodisperse solution

In the main text we report the existence of a new time scale τ_{eq} governing the relaxation of the internal bending modes of polymers in an entangled solution. It is best accessible by observing the time evolution of the mean-square end-to-end distance $\delta R^2(t)$. The equilibration time τ_{eq} is defined as the time at which the internal modes saturate to the value of unconstrained fluctuations $\delta R^2_{eq} = \frac{1}{45} L^4/\ell_p^2$ [12, 17]. In order to analyse how τ_{eq} depends on polymer length L, persistence length ℓ_p and mesh size ξ, we used a procedure analogous to the determination of the entanglement time τ_e: As illustrated in Supplementary Fig. 10, we fitted the intermediate regime of hindered relaxation of $\delta R^2(t)$ at times larger than τ_e with an effective power law, and obtained τ_{eq} by the intersection of that curve with the saturation value δR^2_{eq}.

We have used this procedure to determine τ_{eq} for a broad parameter regime spanning mesh sizes in the range $0.3 \gtrsim \xi/L \gtrsim 0.075$, and persistence lengths in the range $0.2 \lesssim \ell_p/L \lesssim 100$. By varying the polymer stiffness for fixed mesh size, we find that τ_{eq} is independent of ℓ_p; Supplementary Fig. 11 shows a characteristic example for $\xi/L = 0.086$. Recall that we only consider systems with $\xi \ll \ell_p$.

Supplementary Fig. 12 shows τ_{eq} for a set of mesh sizes ξ, and a relative persistence length $\ell_p/L = 1$. We checked several possible scaling laws for the mesh size dependence of τ_{eq}, and found that the best fit is achieved with an exponent close to 2. From the fit of with exponent 2, we also determined the numerical prefactor of τ_{eq} to be

$$\begin{aligned} \tau_{eq} &\approx 0.0016\, \tau_d\, (L/\xi)^2 \\ &= 2.7\times 10^{-4}\, L^5/\xi^2\,. \end{aligned} \tag{3}$$

As a additional test of the scaling law, Eq. (3), we tested how the numerical data for the end-to-end distance fluctuations collapse upon rescaling the time axis as shown in Supplementary Fig. 13. We find excellent data collapse in the whole terminal relaxation regime, further substantiating our scaling law for the equilibration time τ_{eq}.

Scaling in a polydisperse solution

To elucidate the significance of classical constraint release mechanisms, we also studied a system where a single filament of length L (tracer filament) is embedded in an entangled solution of filaments of size L_n, and the same persistence length ℓ_p as the tracer filament. The mesh size ξ of the solution is now defined as $\xi = \sqrt{3/(\nu L_n)}$. Invoking a classical constraint release argument one would argue that constraint release is mediated by filaments (of length L_n) in the entangled solution moving past the tracer filament by curvilinear center of mass motion [18–20]. The corresponding characteristic time scale is given by the disengagement time $\tau_\times^{cr} \sim L_n^3$. However, as shown in Supplementary Fig. 14, in the length range $0.5L_n < L < 3L_n$, the best data collapse of the end-to-end distance fluctuations is achieved by rescaling time t not by a factor proportional to L_n^3 but linear in L_n. These results clearly rule out a classical constraint release mechanisms mediated by curvilinear motion of surrounding chains of length L_n.

Scaling in a solution of polymers with different friction coefficients

The above findings raised the questions: What is or what are the basic time scales that determine the relaxation time of the internal bending modes in an entangled polymer solution? Are the topological constraints mainly released by the motion of the considered filaments or the dynamics of filaments in its surroundings? To answer these questions we considered a chain with friction coefficient ζ (tracer filament) embedded in a solution of filaments whose friction coefficient ζ_n differs from ζ. This enabled us to differentiate between the contribution of the filament's dynamics and the surrounding filaments to the constraint release. In the upper panel of Supplementary Fig. 15 we kept the friction coefficients of the surrounding polymers fixed at $\zeta_n = 1$ and varied the friction coefficient ζ of the tracer polymer. A good data collapse of the tube relaxation time is achieved by rescaling time with $(\zeta_n/\zeta)^{2/3}$, indicating that $\tau_{eq} \sim \zeta^{2/3}$. In contrast, varying ζ_n for fixed $\zeta = 11$ we find good data collapse upon rescaling with a factor $(\zeta_n/\zeta)^{2/3}$; see lower panel of Supplementary Fig. 15. This suggests $\tau_{eq} \sim \zeta_n^{1/3}$. Taken together, these results are consistent with the following scaling form of the equilibration time

$$\tau_{eq} \propto \left(\frac{L}{\xi}\right)^2 \left(\frac{\zeta L^3}{k_B T}\right)^\alpha \left(\frac{\zeta_n L_n^3}{k_B T}\right)^\beta \tag{4}$$

with $\alpha + \beta = 1$. From the data collapse in Supplementary Fig. 15 we estimate that $\alpha \approx 2/3$ and $\beta \approx 1/3$. Both of these exponents for the dependence of the equilibration time on the friction coefficients of the tracer polymer and the surrounding polymers have a certain range of error, and should therefore not be taken too literally. In any case, however, Eq. (4) clearly shows that the equilibration of internal mode results from an interplay between the motion of the tracer filament and its surrounding filaments. Moreover, as the equilibration time is proportional to the square of the number of constraints, $N_\times^2 \sim (L/\xi)^2$, the correlated dynamics of the filament and its surroundings still amounts to a statistically independent release of the topological constraints. However, each of these constraint release events comes about by the correlated motion of the filament and its neighbouring filaments. These correlations are encoded in the exponents of the scaling form of τ_{eq}, Eq. (4).

SUPPLEMENTARY NOTE 4: TERMINAL RELAXATION

We quantified the rotational relaxation by observing the mean-square deviation of the normalized end-to-end vector δe_R^2. Supplementary Fig. 16 shows results from our Brownian dynamics simulations for solutions with different polymer stiffnesses and different mesh sizes, as indicated in the graph. There are three clearly distinct regimes: (1) The *initial relaxation* of a tracer polymer in an entangled solution is identical to the relaxation of a freely diffusing polymer, and given by $\delta e_R^2 \sim t^{3/4}$ (which is due to internal bending modes [4]). (2) This is followed by an *intermediate regime* which coincides with the intermediate regime for the internal modes (δR^2) discussed in the main text: While the rotational relaxation is significantly slowed down as compared to a free polymer it does not come to a halt, again indicating that the Odijk-Semenov tube is only transient. The speed of relaxation, slope of δe_R^2 in the double logarithmic representation of Supplementary Fig. 16, decreases with decreasing mesh size or increasing polymer stiffness. (3) In the final regimes, we find that all curves are well fitted by an expression describing the rotational relaxation of a free rigid rod [4]:

$$\delta e_R^2|_{\text{rigid rod}} = 2 - 2\exp(-t/\tau_r)\,. \tag{5}$$

For a free polymer (red circles in Supplementary Fig. 16), this rigid rod regime is reached for times larger that the relaxation time of internal modes $\sim L^4/\ell_p$. Recently, it has been shown by extensive Brownian dynamics simulations of a two-dimensional system complemented by analytical theory that an effective Perrin theory describes the terminal relaxation dynamics of rigid rods in a dense array of obstacles [21] suggesting that at asymptotically long time scales the dynamics of a rigid rod diffusing in a fixed array is well described by free diffusion though with renormalized diffusion constant parallel and perpendicular to its long axis. Our simulations show that such an effective free diffusion even describes the asymptotic terminal dynamics of a semiflexible polymer in an entangled solution, as implicitly assumed in all previous scaling theories of terminal relaxation [13, 14, 22]. Thus we concluded to use the expression $2 - 2\exp(-t/\tau_r)$ to determine τ_r, and thereby determined the dependence of the terminal relaxation time on polymer stiffness, polymer length and mesh size; see data in the main text.

One of our main conclusions in the main text is that in the semiflexible regime the terminal relaxation time scales as

$$\tau_r \sim \ell_p L^4/\xi^2\,. \tag{6}$$

This is distinct from both Doi's and Odijk's results. To validate it we have performed simulations over a broad parameter range, covering $0.2 \leq \ell_p/L \leq 10$ and $0.075 \leq \xi/L \leq 0.16$. A representative scaling analysis for a typical set of raw data (in the semiflexible regime) is shown in Supplementary Fig. 17. There is a nice data collapse upon rescaling time t by $\tau_r \sim \ell_p L^4/\xi^2$.

SUPPLEMENTARY REFERENCES

[1] Montesi, A., Morse, D. C. & Pasquali, M. Brownian dynamics algorithm for bead-rod semiflexible chain with anisotropic friction. *J. Chem.Phys.* **122**, 84903 (2005).

[2] Lang, P. S. & Obermayer, B. Dynamics of a semiflexible polymer or polymer ring in shear flow. *Phys. Rev. E* **89** (2014).

[3] Kratky, O. & Porod, G. Röntgenuntersuchung gelöster Fadenmoleküle. *Recl. Trav. Chim.* **68**, 1106–1122 (1949).

[4] Edwards, S. F. & Doi, M. *The theory of polymer dynamics* (Clarendon, Oxford, 1986).

[5] Gittes, F. & MacKintosh, F. Dynamic shear modulus of a semiflexible polymer network. *Phys. Rev. E* **58**, 1241–1244 (1998).

[6] Everaers, R., Jülicher, F., Ajdari, A. & Maggs, A. C. Dynamic fluctuations of semiflexible filaments. *Phys. Rev. Lett.* **82**, 3717 (1999).

[7] Morse, D. C. Theory of constrained browninan motion. *Advances in Chemical Physics* **128**, 65–189 (2004).

[8] Farge, E. & Maggs, A. C. Dynamic scattering from semiflexible polymers. *Macromol.* **26**, 5041–5044 (1993).

[9] Frey, E. & Nelson, D. R. Dynamics of flat membranes and flickering in red blood cells. *J. Phys. I (France)* **1**, 1715–1757 (1991).

[10] Granek, R. From semi-flexible polymers to membranes: anomalous diffusion and reptation. *J. Phys. II* **7**, 1761–1788 (1997).

[11] Hallatschek, O., Frey, E. & Kroy, K. Propagation and relaxation of tension in stiff polymers. *Phys. Rev. Lett.* **94**, 77804 (2005).

[12] Kroy, K. & Frey, E. Dynamic scattering from solutions of semiflexible polymers. *Phys. Rev. E* **55**, 3092 (1997).

[13] Odijk, T. The statistics and dynamics of confined or entangled stiff polymers. *Macromol.* **16**, 1340–1344 (1983).

[14] Semenov, A. N. Dynamics of concentrated solutions of rigid-chain polymers. Part 1. Brownian motion of persistent macromol. in isotropic solution. *J. Chem. Soc. Faraday Trans. 2* **82**, 317–329 (1986).

[15] Romanowska, M. *et al.* Direct observation of the tube model in F-actin solutions: Tube dimensions and curvatures. *Europhys. Lett.* **86**, 26003 (2009).

[16] Ramanathan, S. & Morse, D. C. Simulations of dynamics and viscoelasticity in highly entangled solutions of semiflexible rods. *Phys. Rev. E* **76**, 010501 (2007).

[17] Le Goff, L., Hallatschek, O., Frey, E. & Amblard, F. Tracer studies on F-actin fluctuations. *Phys. Rev. Lett.* **89**, 258101 (2002).

[18] Marrucci, G. Relaxation by reptation and tube enlargement: a model for polydisperse polymers. *J. Polym. Sci.: Polym. Phys. Ed.* **23**, 159–177 (1985).

[19] Viovy, J. L., Rubinstein, M. & Colby, R. H. Constraint release in polymer melts: Tube reorganization versus tube dilation. *Macromol.* **24**, 3587–3596 (1991).

[20] McLeish, T. Tube theory of entangled polymer dynamics. *Adv. Phys.* **51**, 1379–1527 (2002).

[21] Munk, T., Höfling, F., Frey, E. & Franosch, T. Effective perrin theory for the anisotropic diffusion of a strongly hindered rod. *Europhys. Lett.* **85**, 30003 (2009).

[22] Doi, M. Effect of chain flexibility on the dynamics of rodlikk polymers in the entangled state. *Journal of Polymer Science: Polymer Symposia* **73**, 93–103 (1985).
