# Supplementary material for: Disentangling entanglements in biopolymer solutions
Source: Nat Commun. 2018 Feb 5;9:494. doi: 10.1038/s41467-018-02837-5 (PMC5799238; doi:10.1038/s41467-018-02837-5)
Supplement: Supplementary file 2 — Description of Additional Supplementary Files [file 41467_2018_2837_MOESM2_ESM.pdf]

Description of Additional Supplementary Files

File Name: Supplementary Movie 1
Description: It shows the time evolution of the full simulation box over a time 25\tau. Initially, the movie runs with 0.000267 \tau per second and is sped up several times by the factors indicated in the movie. For better visibility, a single chain is highlighted in red, whereas the remaining chains are shown in translucent grey. The blue line indicates all positions previously acquired by the center bead of the highlighted chain.

File Name: Supplementary Movie 2
Description: It illustrates the movement of a single chain (red). The hypothetical tube around this tracer chain is shown as a translucent blue-gray chain around the initial position of the polymer. The diameter of the tube is determined by crossover from the free internal relaxation to the intermediate regime, which follows the Odijk scaling. A second tube (yellow-gray) is added at a later time to allow for an easier comparison of tube motion. In total, this video covers a time interval of 0.3 \tau_d.
